# Associations between Fast-Food Restaurants Surrounding Kindergartens and Childhood Obesity: Evidence from China

**DOI:** 10.3390/ijerph18179334

**Published:** 2021-09-03

**Authors:** Chenyang Wang, Zhiping Zhen, Nan Zhao, Chenlin Zhao

**Affiliations:** 1 School of Statistics, Beijing Normal University, Beijing 100875, China; wcy@mail.bnu.edu.cn; 2 Theoretical Teaching and Research Section, College of Physical Education and Sports, Beijing Normal University, Beijing 100875, China; 201821070033@mail.bnu.edu.cn

**Keywords:** childhood obesity, fast-food restaurants, instrumental variable, preschool

## Abstract

The prevalence of obesity among preschool children has risen dramatically due to the influx of Western fast food in China. In this study, we aimed to provide clear evidence on the associations between fast-food restaurants and childhood obesity. We collected and combined three unique cross-sectional datasets: physical fitness data, geographic information, and the financial data of each kindergarten. The two-stage least squares were used for empirical analyses. The final data including 75,730 children were from 785 kindergartens in 82 cities and 23 provinces in China in 2018. The mean age of participants was 4.94 ± 0.87; 34,249 (45.2%) females and 41,481 (54.8%) males. The number of fast-food restaurants within 1, 2, and 3 km radii had a significant and positive correlation with obesity, and this correlation decreased as the radius increased. Furthermore, the distance to the nearest fast-food restaurant had a significant and negative correlation with obesity. Western fast-food restaurants contributed more to obesity than the broader definition of fast-food restaurants. There was marked heterogeneity between urban and rural areas. Our findings documented that fast-food restaurants had a significant and positive association with childhood obesity, thus the restriction of fast-food restaurants surrounding kindergartens might be considered.

## 1. Introduction

Childhood obesity is one of the most serious public health challenges of the 21st century worldwide. The prevalence of obesity and overweight among children, which is not a unique issue in Western developed countries, has risen dramatically in some developing countries, especially China [1]. According to the China Chronic Disease and Nutrition Surveillance 2015–19 survey, the rates of obesity and overweight among children under 6 years old are 3.6% and 6.8%, respectively [2]. As the most populous country in the world, the total number of children younger than 6 years old is enormous in China. Many studies have shown that obesity or overweight in childhood is likely to persist into adulthood [3,4,5,6,7]. Childhood obesity can lead to various health problems, even premature death [8,9,10]. Obesity and overweight have imposed a heavy economic burden on China and induces considerable medical expenditures each year [11,12,13].

Many studies have indicated that the influx of Western fast food in recent decade conveyed health consequences, especially obesity and overweight [14,15,16,17,18,19]. Since the reform and opening-up, China’s economic growth has achieved miraculous levels. With the vigorous development of the market economy, the catering industry has developed rapidly. In addition, income levels and requirements for quality of life have gradually improved. Food-away-from-home has become an intensely popular trend [20,21,22]. Influenced by socioeconomic development and changes in people’s lifestyle, the Chinese food environment has undergone tremendous changes in recent decades. The food environment has a marked influence on individuals’ dietary habits and food consumption, and it is considered to be an important contributor to obesity [23,24,25,26,27,28,29,30,31]. Among all types of restaurants, fast-food restaurants are favored by most people because of their delicious food, convenience, and relatively low prices. On the other hand, fast-food restaurants have received the most criticism. Many children start eating fast food during preschool in China. With exposure to fast-food restaurants, preschool children and their parents are more likely to increase the frequency of eating fast food due to immediate gratification and convenience, leading to obesity and other health problems [32,33]. The plasticity of childhood is great, so early intervention by family and government can prevent the occurrence of obesity and other chronic diseases with time. Therefore, it is crucial to determine the associations between fast-food restaurants and childhood obesity.

Although there are a large number of studies concerning the associations between fast-food restaurants and obesity, they have not all reached the same conclusions [34,35,36,37]. However, many studies have reached the consensus that by using the number and the distance as proxies of the proximity of fast-food restaurants, it may be difficult to avoid the challenge of endogeneity. They typically utilize a feature of the environment, specifically proximity to highways or highway exits, as an instrument. The number and distance are generally measurements of the availability of highways or highway exits. Highway exits are a determinant of the locations of fast-food restaurants; thus, the number of highway exits per county has been used as an instrument [38,39]. Nevertheless, there are some limitations to this method; for instance, some counties do not have any highway exits. Thus, the locations of fast-food restaurants are not determined by highway exits in these counties, and this instrument did not provide any information. Additional studies have used the straight-line distance to the nearest highway as an instrument [4,40,41,42].

An instrument that is valid in one environment may not be valid in others [39]. Employing the proximity of highways as an instrumental variable is reasonable for American studies because the placements of fast-food restaurants are usually around highways. However, the Chinese fast-food industry is entirely different, and the placement of fast-food restaurants is not based on accessibility to the highway. Fast-food restaurants tend to be located in densely populated areas with convenient public transportation. In addition, China has a completely developed public transport system that is convenient for both urban and rural residents. Many people choose to commute by bus because of traditional habits and the low cost of the bus system. Therefore, it is more appropriate to consider the availability of the bus when choosing the instrument in our study. Specifically, we chose the straight-line distance from a kindergarten to the nearest bus stop as an instrumental variable. This instrumental variable is in line with the socioeconomic situation in China. More importantly, it is highly associated with the availability of fast-food restaurants and does not affect other factors that might contribute to obesity. At the same time, we expect that the distance from a kindergarten to the nearest bus stop negatively correlates with the number of fast-food restaurants within a specific radius and positively correlates with the distance to the nearest fast-food restaurant.

There has been some research regarding the associations between the food environment and obesity in different countries, especially focusing on preschool children. Koleilat et al. found that both the number of convenience stores and the number of supermarkets had significant and positive associations with obesity in children 3–4 years of age in Los Angeles County [43]. Salois concluded that the environmental infrastructure was significantly associated with obesity in low-income children 2–4 years of age in the United States (U.S.), and there was heterogeneity between urban and rural areas [44]. Lakes and Burkart demonstrated a positive association between the density of fast-food restaurants and obesity using survey data from children 5–6 years of age in Berlin [45]. There were many samples in additional studies as well, including preschool children [46,47,48,49]. Nevertheless, most studies from China involve children greater than 7 years old, adolescents or adults. Xu et al. found that community exposure to Western fast-food restaurants was positively associated with adults’ body weight status in the rural population, and this association was temporally dynamic [50]. Using cross-sectional data in 2019 and adjusted multiple linear regression, Zhang et al. identified some significant associations between the neighborhood food environment and obesity in older Chinese adults 65 to 80 years of age [51]. In summary, we found no evidence in China on the associations between fast-food restaurants and obesity specifically in preschool children.

Based on the above analysis, there are limitations from three aspects in the existing literature. First, possibly due to a lack of data, there has been little evidence about the associations between fast-food restaurants and obesity in preschool children in China. Second, the challenge of endogeneity is often ignored when the multivariate linear model is exploited in many studies. Third, the samples from China used in most studies come from a given region and are not representative of China as a whole. We filled these gaps and contributed to the existing literature in three aspects. First, we combined three unique cross-sectional datasets from 2018, physical fitness data of 75,730 preschool children, the availability of fast-food restaurants from the Gaode map application programming interface (API), and the financial data of each kindergarten from the Center for Education Economics and Statistics of China to estimate the associations between fast-food restaurant proximity surrounding kindergartens and obesity. Second, combining with the socioeconomic situation in China, we chose the distance from a kindergarten to the nearest bus stop as an instrumental variable to validly address the possible endogeneity problem. Finally, our sample at the individual level comes from 785 kindergartens in 82 cities and 23 provinces in China. These kindergartens are located in the eastern, central, and western regions of China, including areas at all levels of economic development. Therefore, it reflects the general situation in China.

In this study, we estimated the associations between the availability of fast-food restaurants and preschool childhood obesity using two-stage least squares (2SLS). We also analyzed the heterogeneity between urban and rural kindergartens and between females and males. Before this empirical analysis, three hypotheses are proposed:

**Hypothesis** **1** **(H1).** *The number of fast-food restaurants surrounding kindergartens has a positive correlation with childhood obesity*.

**Hypothesis** **2** **(H2).** *The straight-line distance from a kindergarten to the nearest fast-food restaurant has a negative correlation with childhood obesity.*

**Hypothesis** **3** **(H3).** *The correlations between fast-food restaurants differ between urban and rural kindergartens and between females and males.*

## 2. Materials and Methods

### 2.1. Data Collection

We combined three unique data sources. The first one at the individual level came from the College of Physical Education and Sports, Beijing Normal University. The personal characteristics included the height, weight, age and gender of each child. In our sample, height and weight were all measured by professionals, ensuring objectivity and accuracy. The second dataset, including geographic information, was from the Gaode map. The Gaode map provides API on its open platform so that anyone can register an account and use its abundant functions. It contains very comprehensive geographic information, and it is convenient and easy to obtain data. The last data source at the kindergarten level, covering the type, total educational expenditure, building area, number of students, number of teachers and other financial information of each kindergarten, was derived from the Center for Education Economics and Statistics of China. Eventually, we obtained the final cross-sectional data, including 75,730 children 3–6 years of age from 785 kindergartens in 82 cities and 23 provinces in China in 2018 for this study.

### 2.2. Obesity Outcomes

Although there are many techniques for measuring obesity, body mass index (BMI) is widely used [52]. BMI is calculated using the following formula: BMI = weight (kg)/height^2^ (m^2^). Different organizations have different definitions of obesity and overweight. Cutoffs from the World Health Organization (WHO), International Obesity Task Force (IOTF) and the Centers for Disease Control and Prevention (CDC) are generally used [53,54]. In the subsequent empirical analysis, we defined childhood obesity for each child using cutoffs from the WHO. Whether a child is considered obese or overweight is always determined by the Child Growth Standards according to different organizations. For instance, a child over 5 years, with a BMI value greater than 2 standard deviations (SDs) above the WHO Child Growth Standards median, is considered obese [55]. For the explained variable, we set a dummy variable representing whether a child was obese according to cutoffs from the WHO. Subsequently, we used the standard developed by the IOTF and CDC to test the robustness of the models.

### 2.3. The Definitions of Fast-Food Restaurants

Defining fast-food restaurants and the proximity of fast-food restaurants is crucial before estimating the associations between fast-food restaurants and obesity. There are no strict definitions of fast-food restaurants in China, which are generally divided into Western fast-food restaurants and Chinese fast-food restaurants [22,56]. Western fast-food restaurants generally sell hamburgers, pizzas, fried chicken, and other food that can be prepared and served quickly. Western fast-food chains with brands are the most popular in China and predominantly come from the U.S., such as Kentucky Fried Chicken (KFC), McDonald’s and Pizza Hut. The Chinese fast-food restaurant is a relative concept to the Western fast-food restaurant. Based on Chinese dietary habits, Chinese fast-food restaurants usually serve a wider variety of foods, such as rice, noodles or rice noodles, and pastries, such as pancakes and dumplings. Influenced by the rapid growth of China’s economy and the astonishing success of the Western fast-food industry, the Chinese fast-food industry has grown rapidly due to its low cost of establishment and flexible operating mode. Different from Western fast-food restaurants, most Chinese fast-food restaurants are self-employment businesses.

There are generally two strategies for defining which establishments to categorize as fast-food restaurants that have been used in previous studies. The first is selecting several specific fast-food brands. A number of studies followed Currie et al. [57], choosing the top 10 chains from Dun and Bradstreet according to popularity or from Wikipedia according to the total number. The second strategy uses a broader definition that includes several types of restaurants, including the first definition of fast-food restaurants. For example, Anderson and Matsa adopted the definition from the United States Census Zip Code Business Patterns for the classification of restaurants [40]. They defined fast-food restaurants as both full-service and limited-service restaurants. Asirvatham et al. directly defined fast-food restaurants as hamburger fast-food restaurants, sandwich places, and pizzerias [42]. Based on the definitions of the existing literature, we also defined fast-food restaurants in two ways. First, following Currie et al. [57], we used the top 10 western fast-food brands, denoted as Top, with the largest total number of locations in China. We collected the data from the official websites of each brand. The final list was Wallace, KFC, McDonald’s, Pizza Hut, Dicos, Champion Pizza, Burger King, Subway, Domino’s Pizza, and Papa John’s. Second, following Anderson and Matsa and considering the specific situation of the fast-food industry in China, we used a broader definition that includes all types of fast food, denoted as FF. In the subsequent empirical analyses, we compared the results using two definitions (Top and FF).

Defining a proxy of the proximity of fast-food restaurants is also an essential premise for studying the associations between fast-food restaurants and obesity. In summary, there are two main types of proxies: the total number within a specific radius and the distance to the nearest fast-food restaurant [40,41,42,50,57]. Theoretically, the choice of the measurement of the proximity of fast-food restaurants should not influence the results of our study. Thus, we used the two measurements in our major empirical analyses.

### 2.4. Control Variables

We controlled for factors at the kindergarten level of each child in our models. The final dataset had a total of 785 kindergartens’ information. All kindergartens in the sample are divided into two types, those located in the city and those located in the countryside. Given the unique dual economic structure in China, children in urban and rural areas might differ dramatically. Therefore, we controlled the type of kindergarten in our models. To avoid omitting factors as much as possible, we also controlled the total education expenditure per student, the building area per student, and the number of teachers for each kindergarten. We did not have information about each child’s parents or family status, and these kindergarten level control variables may reflect the children’s family socioeconomic status to some extent.

### 2.5. Analysis

#### 2.5.1. Ordinary Least Squares

In our primary analysis, we tested the first two hypotheses raised in the prior section using the entire sample. We first examined the associations between the proximity of fast-food restaurants and childhood obesity using ordinary least squares (OLS). Based on the cross-sectional data at the individual level in 2018, the model is as follows:

Obesity_ijc_ = α_0_ + α_1_ FastFood_jc_ + α_2_ X_ijc_ + α_3_ K_jc_ + μ_c_ + ε_ijc_ (1)

where Obesity is the dependent variable representing preschool childhood obesity outcome. According to the cutoffs from the WHO, Obesity is a dummy variable representing whether the child is obese. FastFood is the key independent variable, representing the proximity of fast-food restaurants to each kindergarten. As mentioned in the previous section, the fast-food restaurant here includes two definitions, Top and FF. The first proxy of the proximity of Top and FF is the number within 1, 2, and 3 km radii (Top1 km, Top2 km, Top3 km, FF1 km, FF2 km, and FF3 km). The second is the straight-line distance from a kindergarten to the nearest Top and FF (disTop, disFF). X and K are control variables. X is a vector of each child’s demographic variables, including age and gender. K is a vector representing kindergarten-level information, including the type of each kindergarten, the total education expenditure per student, the building area per student, and the number of teachers at each kindergarten. We set a dummy variable representing a kindergarten in urban or rural areas. In addition, we controlled the county fixed effects, μ_c_. ε_ijc_ are error terms, which are clustered at the kindergarten level. To reduce problems due to heteroscedasticity we used natural logarithms for all key explanatory variables, total educational expenditure per student, building area per student and number of teachers. Subscripts i, j, and c represent child (i), kindergarten (j), and county (c).

#### 2.5.2. 2SLS

The challenge of endogeneity problems is inescapable in Equation (1). The endogeneity in our study possibly comes from two sources. (1) Missing variables; Obesity is comprehensively determined by many complicated determinants. However, due to the lack of data, we do not have information on each child’s parents and families, such as family income, parents’ education level and eating patterns and habits. (2) Self-selection problem: Obese or overweight children may be more predisposed to fast-food than others. Instrumental variable methods are valid and common to solve this endogeneity problem. As mentioned before, keeping the control variables unchanged, we exploited 2SLS to estimate the adjusted model. The final models of two stages are as follows:

FastFood_jc_ = β_0_ + β_1_ disbus_jc_ + β_2_ X_ijc_ + β_3_ K_jc_ + μ_c_ + δ_ijc_, (2)

(3) $${\mathrm{Obesity}}_{\mathrm{ijc}}=\gamma_{0}+\gamma_{1}\;\hat{{\mathrm{FastFood}}_{\mathrm{jc}}}+\gamma_{2}\;X_{\mathrm{ijc}}+\gamma_{3}\;K_{\mathrm{jc}}+\mu_{c}+\sigma_{\mathrm{ijc}},$$

where disbus is the straight-line distance from a kindergarten to the nearest bus stop and is taken as the natural logarithm. $\hat{\mathrm{FastFood}}$ is the fitted value of the first stage. The other variables have the same meaning as in Equation (1) and county fixed effects are controlled. δ_ijc_ and σ_ijc_ are error terms of the first and second stages, respectively. They are both clustered at the kindergarten level.

## 3. Results

### 3.1. Descriptive Statistics

**Figure 1:**
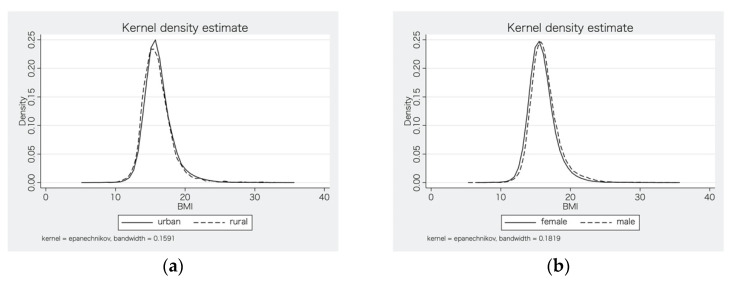
(**a**) Kernel density curves of urban and rural subsamples; (**b**) kernel density curves of female and male subsamples.

The final cross-sectional sample included a total of 75,730 children from 785 kindergartens, 224 counties, 85 cities, and 23 provinces. The descriptive statistical analysis is shown in Table 1. The obesity rate of the sample was as high as 7.08%, and 45.23% of the children were girls. The mean age of the children was 4.94 years, and they were predominately from urban kindergartens (94.40%). Panel (a) and (b) of Figure 1 show the kernel density curves of urban and rural, female and male subsamples, respectively. In terms of overall distribution, children in the urban sample were fatter than those in the rural sample, and males were fatter than females. The minimums of the number of fast-food restaurants were all 0.

### 3.2. OLS and 2SLS

The OLS results are shown in Table 2. As shown in columns 5–7 in Table 2, the estimated coefficients of the number of FFs within 1, 2, and 3 km were all statistically significant at the 5% level. However, the values of the estimated coefficients, likely biased, were very small. Nevertheless, all the key independent variables in the other models were insignificant. In general, there was no solid evidence that the proximity of Top or FF had a positive correlation with childhood obesity. The results of OLS were not in line with our prior expectations.

**Figure 2:**
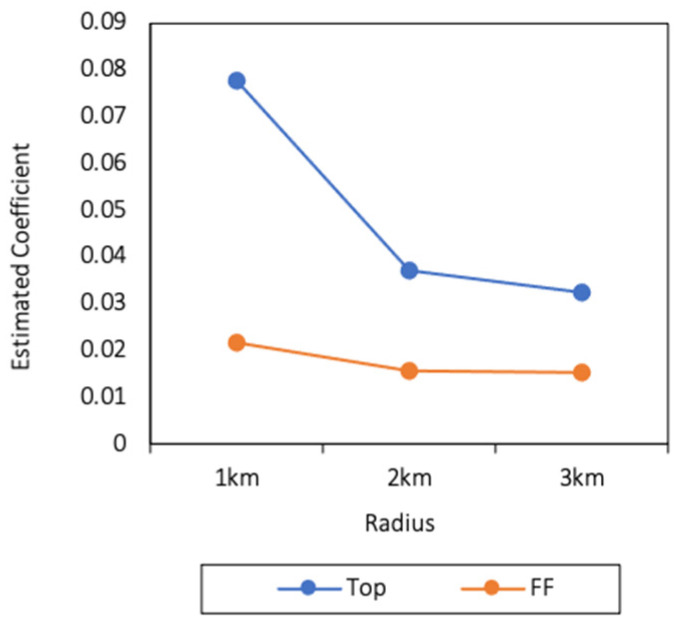
Estimated coefficients of the number of Tops and FFs within 1, 2, 3 km by 2SLS.

In contrast, the 2SLS results were completely different. As shown in Table 3. the estimated coefficients of the number of Tops and FFs within 1, 2, and 3 km and the distance to the nearest Top and FF were all statistically significant at the 1% level. The estimated coefficients of the number of Tops and FFs within 1, 2, and 3 km are plotted in Figure 2. Interestingly, in Figure 2, the estimated coefficients of the number of Tops decreased when the radius increased, and the values of the estimated coefficients of the number of FFs showed the same pattern. Furthermore, the values of the estimated coefficients of the number of Tops were always much larger than the number of FFs when the radius was the same. Our findings demonstrate that fast-food restaurants have an association with childhood obesity and that this association wanes as the distance increases. In addition, Western fast-food restaurants contribute more to childhood obesity.

As described in the previous section, there were potential endogeneity problems. The comparison between the results of OLS and 2SLS confirmed this hypothesis. Employing the distance from each kindergarten to the nearest bus stop as an instrument, we theoretically addressed endogeneity problems. Using 2SLS to estimate the coefficients of all models, we separated out the exogenous parts caused by the instrumental variable to acquire consistent estimates. The results of the first stage are shown in Table 4. We tested the endogeneity of the instrument using the Durbin–Wu–Hausman test (DWH). The third row from the bottom in Table 4 shows the *p*-values of DWH, which were all less than 1%. This indicated that the two proxies of the proximity of fast-food restaurants we used in this study exhibited endogeneity. The second row from the bottom in Table 4 shows that the F-statistics in the first stages of all models were larger than 16, which was indicative of a high correlation between the proximity of fast-food restaurants and the instrument. Specifically, the instrument was negatively correlated with the number of fast-food restaurants and positively correlated with the distance to the nearest fast-food restaurant at the 1% level. In summary, the results of 2SLS were consistent with our expectations, and the instrument passed all tests.

### 3.3. Robustness

We used two methods to determine robustness. First, for the dependent variable, we reset a dummy variable according to the criteria of overweight defined by the WHO. That is, the dependent variable (overw) is equal to 1 when the child is overweight. The results of the second stage are shown in Table 5. A second robustness test executed the different cutoffs of obesity. We used criteria published by IOTF and CDC to set a dummy as the dependent variable (obIOTF and obCDC). Theoretically, the criteria of obesity should not impact the conclusions. The second results are shown in Table 5.

As shown in Table 5, the three main conclusions hold. (1) Fast-food restaurants have a significant and positive association with childhood obesity. (2) This association decreases when the radius increases. (3) Western fast-food restaurants contribute more to childhood obesity.

### 3.4. Heterogeneity

Next, we analyzed the heterogeneity between the two types of kindergartens and gender. There are already some studies acknowledging that heterogeneity does exist in groups with different geographical features or demographic characteristics. Due to the unique dual economic structure in China, the commercial economy in urban areas may be more developed. There are more fast-food restaurants in urban areas in China. Thus, urban preschool children are more exposed to fast-food restaurants than children in rural areas. Moreover, in regard to obesity issues, the heterogeneity between females and males cannot be ignored. Therefore, we divided the whole sample according to two types of kindergartens and gender. Then, we examined the associations between fast-food restaurants and childhood obesity by 2SLS using the four subsamples separately.

The results of the urban and rural subsamples are shown in Table 6. As shown in Table 6, the estimated coefficients of the proximity of fast-food restaurants were statistically significant, indicating that the main conclusions of this study appeared to hold in the urban subsample. Nevertheless, in the rural subsample, all estimated coefficients of the key variables were insignificant, which showed that the mechanism was invalid in the rural area for preschool children. The different results in the two subsamples shown in Table 6 confirmed the existence of heterogeneity between urban and rural areas, which supported the third hypothesis we raised. This heterogeneity is likely due to different lifestyles and dietary habits between urban and rural areas, and preschool children in urban areas are more likely to be influenced by fast-food restaurants.

The difference between females and males is displayed in Table 7. The results of the two subsamples divided by gender were similar to the results of the whole sample, and almost all conclusions held. For both the female and male subsamples, the results of key independent variables were all statistically significant at the 1% level. The values of the estimated coefficients were close between the two subsamples, which reflected that the influential mechanism between fast-food restaurants and preschool childhood obesity was similar in female and male children. This conclusion was not consistent with our hypothesis.

**Figure 3:**
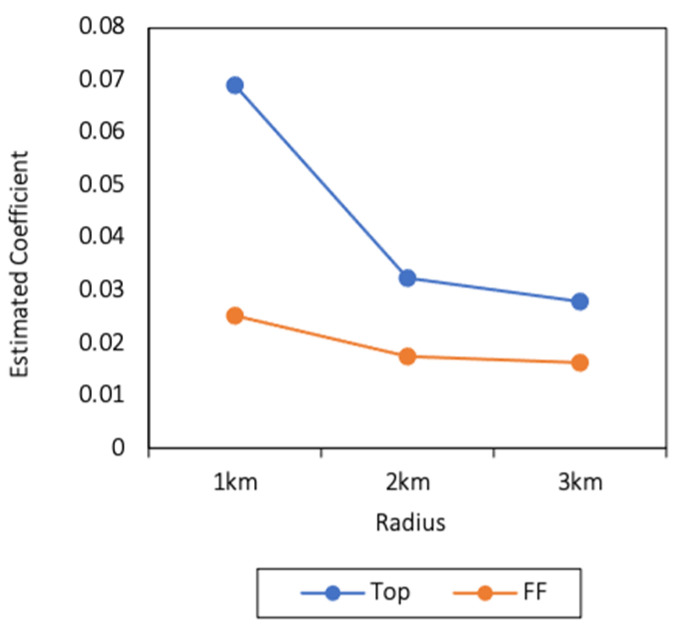
Estimated coefficients of the number of Tops and FFs within 1, 2, and 3 km of the urban subsample.

**Figure 4:**
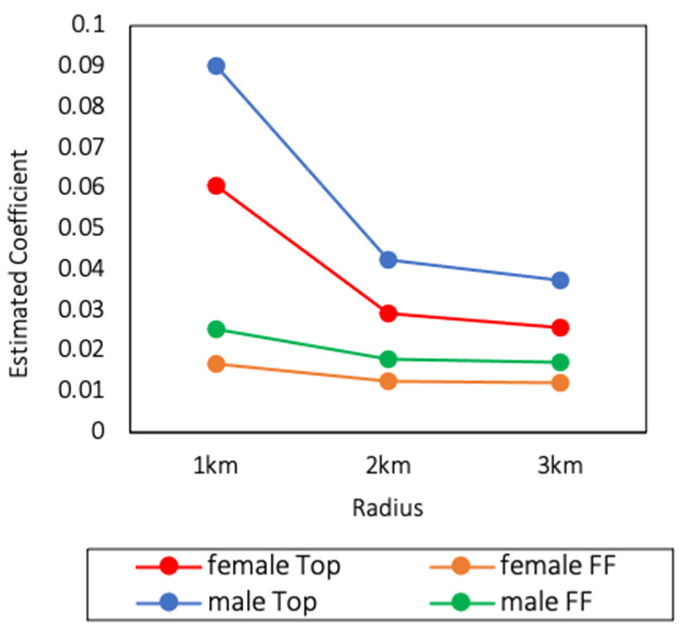
Estimated coefficients of the number of Tops and FFs within 1, 2, and 3 km of the female and male subsample.

Consistent with the whole sample, the correlation between the number of Western fast-food restaurants and childhood obesity was even greater. Specifically, the values of the estimated coefficients of the number of Tops are always much larger than the number of FFs in the subsamples. Since the estimated coefficients of the number of Tops and FFs within 1, 2, and 3 km of the rural subsample were insignificant, the results of the urban subsample were plotted as shown in Figure 3. The results of the female and male subsamples are plotted in Figure 4. As we can see, the Western fast-food restaurants affected childhood obesity more from the number aspect. In general, the three primary conclusions of this article appeared to hold in the urban, female and male subsamples.

## 4. Discussion

Our study contributes to the existing literature with firm evidence that fast-food restaurants have a positive correlation with obesity in China. Robust findings appeared after the potential endogeneity problem was addressed. We also identified heterogeneity between urban and rural kindergartens.

First, we combined three unique data sources, which are all for preschool. In general, most Chinese studies about health issues use data from the China Health and Nutrition Survey (CHNS) [18,50,58]. However, due to confidentiality, it is impossible to obtain specific information at the individual level, such as the residential address or name of the school. Some studies have also used data at the individual level by questionnaires or surveys. However, the data are generally from one region, and the results are not generalizable to all of China [13,51]. Our data are from 85 cities and 23 provinces, which reflects the universal situation in China. Our study specifically focused on preschool children, which filled the gaps about the associations between fast-food restaurants and obesity in preschool children. In addition, all our datasets are official and objective, providing specific and solid evidence.

Second, we used two proxies of the proximity of fast-food restaurants: the number of fast-food restaurants within a specific radius and the straight-line distance to the nearest fast-food restaurant. These are direct and valid for measuring the availability of fast-food restaurants surrounding each kindergarten. More importantly, changing the proxy variable did not affect the positive associations between of fast-food restaurants and obesity, which was in line with our expectations.

Third, we exploited the 2SLS method to address the potential endogeneity problem by using an instrument. This instrument, the distance to the nearest bus stop, is a proxy of the proximity of public transportation and passes all instrumental variable tests. Different from previous American studies using the proximity of highways as an instrument [4,38,39,40,41,42], our choice is based on the fact that China has a completely developed public transport system, and fast-food restaurants tend to be located in places that are easily accessible. Therefore, instead of using the proximity of highways as an instrument such as in previous American studies, using bus stops proximity is more appropriate in China.

Fourth, the comparison between the results of OLS and 2SLS indicates some fascinating findings. The results of OLS show that fast-food restaurants have no statistical correlations with preschool childhood obesity. In contrast, the result entirely changed once the potential endogeneity was addressed, and these results were always robust. Fast-food restaurants have a positive and significant association with obesity, which is consistent with Lakes and Burkart [45]. Compared with previous studies, our findings are consistent with Asirvatham et al. [42], indicating that this association decreases as the distance increases. Using two definitions of fast-food restaurants, we found that Western fast-food restaurants exerted a greater influence on obesity than the broader definition.

Moreover, many studies have considered heterogeneity and reported different results in subsamples [39,44]. We considered the heterogeneity between different types of kindergarten and gender. The results of the urban subsamples were similar to those of the whole sample, but the correlations between fast-food restaurants and obesity were not statistically significant in the rural subsample. This difference between urban and rural kindergartens indicates that preschool children in urban areas are more sensitive to fast-food restaurants. The correlations between fast-food restaurants and childhood obesity are all significant in both the female and male subsamples, and the values of the estimated coefficients were slightly different between females and males. Therefore, the correlation between fast-food restaurants and obesity is different only between urban and rural areas for preschool children.

Regrettably, there are several limitations in our study. First, we did not have access to comprehensive, time-varying data. Thus, we were unable to capture the dynamic trend of how the associations between fast-food restaurants and childhood obesity change over time. These problems are addressed later when panel data are available. Second, due to the lack of data on eating patterns and consumption behaviors, we could not analyze the deeper cause and mechanism of the effect of fast-food restaurants on obesity. Third, we did not obtain information on the family status of each child to control for the factors of the parents. Using the financial data that were available at the kindergarten level, we addressed this problem to some extent.

Finally, there have been no laws or regulations restricting the distribution of fast-food restaurants surrounding kindergartens in China yet. However, our findings confirm that a positive association between fast-food restaurants, especially Western fast-food restaurants, and preschool childhood obesity does exist. This provides policymakers the inspiration that appropriate regulations are possibly necessary and might need to limit the proximity of fast-food restaurants surrounding kindergartens. Furthermore, infrastructure construction can be strengthened, such as constructing more parks or playgrounds around kindergartens. Health behavioral guidance should be provided to parents to prevent the prevalence of chronic diseases as early as possible.

## 5. Conclusions

This study estimated the associations between fast-food restaurants and preschool childhood obesity using 2SLS and reached three conclusions. First, our findings document that fast-food restaurants have a significant and positive association with obesity. Specifically, the number of fast-food restaurants within 1, 2, and 3 km radii has a significant and positive correlation with obesity, and this correlation decreases as the radius increases. Furthermore, the distance to the nearest fast-food restaurant has a significant and negative correlation with obesity. Second, Western fast-food restaurants contribute more to obesity than the broader definition of fast-food restaurants. Third, there is marked heterogeneity between urban and rural kindergartens but not between females and males. This suggests that parents need to pay more attention to preschool children’s diet and obesity to prevent chronic diseases, and policymakers may consider the distribution of fast-food restaurants around kindergartens.

## Figures and Tables

**Table 1:** Descriptive statistics.

Variables	N (%)	Mean (S.D.)	Min	Max
Obesity				
Yes	5365 (7.08)			
No	70,365 (92.92)			
Gender				
Female	34,249 (45.23)			
Male	41,481 (54.77)			
Type of each kindergarten				
Urban	71,490 (94.40)			
Rural	4240 (5.60)			
BMI (kg/m^2^)		16.14 (2.08)	5.30	35.48
Age (year)		4.94 (0.87)	3	6.92
Number of Top within 1 km		2.17 (2.66)	0	16
Number of Top within 2 km		7.12 (11.16)	0	129
Number of Top within 3 km		13.83 (24.37)	0	274
Distance to the nearest Top (m)		2652.14 (5566.40)	23	50,001
Number of FF within 1 km		59.95 (59.76)	0	347
Number of FF within 2 km		230.33 (205.67)	0	879
Number of FF within 3 km		404.70 (310.16)	0	893
Distance to the nearest FF (m)		240.93 (463.45)	2	7619
Building area per student (m^2^)		11.07 (11.97)	1.83	137.89
Total education expenditure per student (yuan)		8202.59 (11,507.51)	721.56	150,301.30
Number of teachers		140.43 (332.26)	10	3465

**Table 2:** Results of OLS.

	(1)	(2)	(3)	(4)	(5)	(6)	(7)	(8)
Dependent	Obesity	Obesity	Obesity	Obesity	Obesity	Obesity	Obesity	Obesity
Independent	Top1 km	Top2 km	Top3 km	disTop	FF1 km	FF2 km	FF3 km	disFF
	0.004	0.004	0.003	−0.002	0.006 ***	0.005 **	0.004 **	−0.003
	(0.004)	(0.003)	(0.003)	(0.002)	(0.002)	(0.002)	(0.002)	(0.002)
Control variable	Yes	Yes	Yes	Yes	Yes	Yes	Yes	Yes
County fixed effect	Yes	Yes	Yes	Yes	Yes	Yes	Yes	Yes
N	75,730	75,730	75,730	75,730	75,730	75,730	75,730	75,730

Note: Significance levels *** *p* < 0.01, ** *p* < 0.05, All key independent variables were taken natural logarithms, and robust standard errors clustered at the kindergarten level appeared in parentheses.

**Table 3:** Results of 2SLS.

	(1)	(2)	(3)	(4)	(5)	(6)	(7)	(8)
Dependent	Obesity	Obesity	Obesity	Obesity	Obesity	Obesity	Obesity	Obesity
Independent	Top1 km	Top2 km	Top3 km	disTop	FF1 km	FF2 km	FF3 km	disFF
	0.078 ***	0.037 ***	0.033 ***	−0.023 ***	0.022 ***	0.016 ***	0.015 ***	−0.033 ***
	(0.028)	(0.011)	(0.010)	(0.007)	(0.006)	(0.004)	(0.004)	(0.010)
Control variable	Yes	Yes	Yes	Yes	Yes	Yes	Yes	Yes
County fixed effect	Yes	Yes	Yes	Yes	Yes	Yes	Yes	Yes
N	75,730	75,730	75,730	75,730	75,730	75,730	75,730	75,730

Note: Significance levels *** *p* < 0.01, All key independent variables were taken natural logarithms, and robust standard errors clustered at the kindergarten level appeared in parentheses.

**Table 4:** Results of the first stage.

	(1)	(2)	(3)	(4)	(5)	(6)	(7)	(8)
Dependent	Top1 km	Top2 km	Top3 km	disTop	FF1 km	FF2 km	FF3 km	disFF
Independent	disbus	disbus	disbus	disbus	disbus	disbus	disbus	disbus
	−0.103 ***	−0.217 ***	−0.246 ***	0.350 ***	−0.369 ***	−0.513 ***	−0.527 ***	0.245 ***
	(0.025)	(0.037)	(0.043)	(0.056)	(0.060)	(0.066)	(0.064)	(0.050)
Control variable	Yes	Yes	Yes	Yes	Yes	Yes	Yes	Yes
County fixed effect	Yes	Yes	Yes	Yes	Yes	Yes	Yes	Yes
Endogeneity test	0.001	0.001	0.001	0.000	0.000	0.000	0.006	0.000
F-statistics	16.864	34.757	32.495	39.190	38.351	59.574	67.322	24.008
N	75,730	75,730	75,730	75,730	75,730	75,730	75,730	75,730

Note: Significance levels *** *p* < 0.01, All dependent variables and the instrument were taken natural logarithms, and robust standard errors clustered at the kindergarten level appeared in parentheses.

**Table 5:** Robustness.

	(1)	(2)	(3)	(4)	(5)	(6)	(7)	(8)
Dependent	overw	overw	overw	overw	overw	overw	overw	overw
Independent	Top1 km	Top2 km	Top3 km	disTop	FF1 km	FF2 km	FF3 km	disFF
	0.119 **	0.056 ***	0.050 ***	−0.035 ***	0.033 ***	0.024 ***	0.023 ***	−0.050 ***
	(0.046)	(0.019)	(0.017)	(0.012)	(0.011)	(0.008)	(0.007)	(0.018)
Control variable	Yes	Yes	Yes	Yes	Yes	Yes	Yes	Yes
County fixed effect	Yes	Yes	Yes	Yes	Yes	Yes	Yes	Yes
N	75,730	75,730	75,730	75,730	75,730	75,730	75,730	75,730
Dependent	obIOTF	obIOTF	obIOTF	obIOTF	obIOTF	obIOTF	obIOTF	obIOTF
Independent	Top1 km	Top2 km	Top3 km	disTop	FF1 km	FF2 km	FF3 km	disFF
	0.054 **	0.025 ***	0.023 ***	−0.160 **	0.015 ***	0.011 ***	0.011 ***	−0.023 **
	(0.024)	(0.010)	(0.009)	(0.006)	(0.006)	(0.004)	(0.004)	(0.009)
Control variable	Yes	Yes	Yes	Yes	Yes	Yes	Yes	Yes
County fixed effect	Yes	Yes	Yes	Yes	Yes	Yes	Yes	Yes
N	75,730	75,730	75,730	75,730	75,730	75,730	75,730	75,730
Dependent	obCDC	obCDC	obCDC	obCDC	obCDC	obCDC	obCDC	obCDC
Independent	Top1 km	Top2 km	Top3 km	disTop	FF1 km	FF2 km	FF3 km	disFF
	0.083 **	0.040 ***	0.035 ***	−0.024 **	0.023 ***	0.017 ***	0.016 ***	−0.035 **
	(0.036)	(0.015)	(0.013)	(0.010)	(0.008)	(0.006)	(0.006)	(0.014)
Control variable	Yes	Yes	Yes	Yes	Yes	Yes	Yes	Yes
County fixed effect	Yes	Yes	Yes	Yes	Yes	Yes	Yes	Yes
N	75,730	75,730	75,730	75,730	75,730	75,730	75,730	75,730

Note: Significance levels *** *p* < 0.01, ** *p* < 0.05, All key independent variables were taken natural logarithms, and robust standard errors clustered at the kindergarten level appeared in parentheses.

**Table 6:** Urban and rural subsamples.

	(1)	(2)	(3)	(4)	(5)	(6)	(7)	(8)
Results of Urban Subsamples.								
Dependent	Obesity	Obesity	Obesity	Obesity	Obesity	Obesity	Obesity	Obesity
Independent	Top1 km	Top2 km	Top3 km	disTop	FF1 km	FF2 km	FF3 km	disFF
	0.069 **	0.032 ***	0.028 ***	−0.024 ***	0.025 ***	0.017 ***	0.016 ***	−0.033 ***
	(0.027)	(0.032)	(0.009)	(0.008)	(0.008)	(0.006)	(0.005)	(0.012)
Control variable	Yes	Yes	Yes	Yes	Yes	Yes	Yes	Yes
County fixed effect	Yes	Yes	Yes	Yes	Yes	Yes	Yes	Yes
N	71,490	71,490	71,490	71,490	71,490	71,490	71,490	71,490
Results of rural subsamples.								
Dependent	Obesity	Obesity	Obesity	Obesity	Obesity	Obesity	Obesity	Obesity
Independent	Top1 km	Top2 km	Top3 km	disTop	FF1 km	FF2 km	FF3 km	disFF
	−0.029	−0.085	−0.035	0.014	−0.022	0.560	0.080	−0.027
	(0.047)	(−0.528)	(−0.530)	(0.651)	(−0.889)	(0.074)	(0.324)	(−0.427)
Control variable	Yes	Yes	Yes	Yes	Yes	Yes	Yes	Yes
County fixed effect	Yes	Yes	Yes	Yes	Yes	Yes	Yes	Yes
N	4240	4240	4240	4240	4240	4240	4240	4240

Note: Significance levels *** *p* < 0.01, ** *p* < 0.05, All key independent variables were taken natural logarithms, and robust standard errors clustered at the kindergarten level appeared in parentheses.

**Table 7:** Female and male subsamples.

	(1)	(2)	(3)	(4)	(5)	(6)	(7)	(8)
Results of Female Subsamples.								
Dependent	Obesity	Obesity	Obesity	Obesity	Obesity	Obesity	Obesity	Obesity
Independent	Top1 km	Top2 km	Top3 km	disTop	FF1 km	FF2 km	FF3 km	disFF
	0.061 **	0.029 ***	0.026 ***	−0.018 ***	0.017 ***	0.012 ***	0.012 ***	−0.025 ***
	(0.025)	(0.010)	(0.009)	(0.007)	(0.006)	(0.004)	(0.004)	(0.009)
Control variable	Yes	Yes	Yes	Yes	Yes	Yes	Yes	Yes
County fixed effect	Yes	Yes	Yes	Yes	Yes	Yes	Yes	Yes
N	34,249	34,249	34,249	34,249	34,249	34,249	34,249	34,249
Results of male subsamples.								
Dependent	Obesity	Obesity	Obesity	Obesity	Obesity	Obesity	Obesity	Obesity
Independent	Top1 km	Top2 km	Top3 km	disTop	FF1 km	FF2 km	FF3 km	disFF
	0.090 ***	0.042 ***	0.037 ***	−0.027 ***	0.025 ***	0.018 ***	0.017 ***	−0.038 ***
	(0.033)	(0.013)	(0.012)	(0.008)	(0.007)	(0.005)	(0.005)	(0.012)
Control variable	Yes	Yes	Yes	Yes	Yes	Yes	Yes	Yes
County fixed effect	Yes	Yes	Yes	Yes	Yes	Yes	Yes	Yes
N	41,481	41,481	41,481	41,481	41,481	41,481	41,481	41,481

Note: Significance levels *** *p* < 0.01, ** *p* < 0.05, All key independent variables were taken natural logarithms, and robust standard errors clustered at the kindergarten level appeared in parentheses.

## Data Availability

The data are not publicly available due to ethical reasons.
